# High-performance work systems and innovative work behavior: the mediating role of knowledge hiding and the moderating role of performance goal orientation

**DOI:** 10.3389/fpsyg.2026.1871686

**Published:** 2026-09-24

**Authors:** Jiarui Zhu, Hyuneung Lee

**Affiliations:** Department of Business Administration, College of Economics and Management, Chungnam National University, Daejeon, Republic of Korea

**Keywords:** high-performance work systems, innovative work behavior, knowledge hiding, performance goal orientation, social exchange theory

## Abstract

Although high-performance work systems (HPWS) are widely recognized as an important driver of employee performance, less is known about how they may restrain defensive knowledge behaviors that ultimately shape innovation outcomes. Drawing on an integrated framework combining the AMO model, social exchange theory, and goal orientation theory, this study develops and tests a moderated mediation model in which HPWS are associated with innovative work behavior (IWB) through knowledge hiding, with performance goal orientation (PGO) serving as a boundary condition on the relationship between HPWS and knowledge hiding. Using survey data from 456 full-time employees working in China, the results indicate that employees’ perceptions of HPWS are negatively associated with knowledge hiding and positively associated with IWB, whereas knowledge hiding is negatively associated with IWB. Moreover, PGO strengthened the negative relationship between HPWS and knowledge hiding, such that this negative relationship is more pronounced at higher levels of PGO. Consistent with this pattern, the indirect positive association between HPWS and IWB via reduced knowledge hiding is stronger when employees report higher levels of PGO. By integrating the resource, relational, and self-regulatory processes through which employees respond to HPWS, this study extends research on strategic human resource management, knowledge management, and employee innovation.

## Introduction

Organizations increasingly rely on employees’ innovative work behavior (IWB), commonly defined as the generation, promotion, and implementation of new ideas to sustain competitiveness in dynamic environments (de Jong and den Hartog, 2010; Janssen, 2000; Scott and Bruce, 1994). Because innovation unfolds through a multistage process, employees’ IWB is highly sensitive to organizational conditions that either enable or constrain idea development and implementation. In this sense, understanding the organizational antecedents of IWB remains central to both strategic human resource management and innovation research.

Within this stream, high-performance work systems (HPWS) have long been regarded as a critical organizational mechanism for eliciting discretionary effort and enhancing employee performance. As internally coherent bundles of HR practices, HPWS typically include selective staffing, extensive training, performance-based rewards, participation, and information sharing, all of which are expected to enhance employees’ ability, motivation, and opportunity to contribute (Appelbaum et al., 2000; Huselid, 1995; Jiang et al., 2012). Recent employee-level studies identify perceived organizational support (Kim et al., 2023), social capital and knowledge sharing (Arshad et al., 2024), and work engagement and knowledge sharing (Escribá-Carda et al., 2025) as enabling mechanisms linking HPWS to innovative behavior. Related evidence further identifies employee creativity as a mediator and knowledge sharing as a moderator of the HPWS–IWB relationship (Amoozegar et al., 2025). However, these studies primarily emphasize enabling mechanisms, leaving comparatively less understood whether HPWS may also promote IWB by reducing knowledge hiding, a deliberate behavioral barrier to knowledge exchange. This omission is important because innovation depends not only on employees’ capability and motivation but also on whether they are willing to exchange and mobilize knowledge rather than strategically withhold it. A particularly relevant yet often overlooked impediment to innovation is knowledge hiding, defined as the intentional attempt to withhold or conceal requested knowledge from others (Connelly et al., 2012). Importantly, knowledge hiding is conceptually distinct from the mere absence of knowledge sharing. Whereas knowledge sharing concerns whether employees provide useful information, knowledge hiding captures a deliberate decision to withhold knowledge in response to a direct request. This distinction is theoretically meaningful because the motives, relational implications, and organizational consequences of intentional concealment differ from those associated with passive non-sharing.

From a social exchange perspective, knowledge hiding is especially damaging because it violates expectations of reciprocity, mutual support, and cooperation (Blau, 1964). When employees intentionally conceal knowledge, they signal distrust and weaken relational norms, thereby discouraging future knowledge exchange and undermining collaborative ties. By contrast, the absence of knowledge sharing may arise from situational constraints such as limited time, lack of awareness, or uncertainty about the usefulness of one’s knowledge, and therefore does not necessarily produce the same relational damage. Because IWB depends heavily on timely access to task-relevant information, iterative feedback, and the integration of dispersed expertise, the deliberate concealment of requested knowledge is likely to be particularly detrimental to innovation. Consistent with this view, prior research has linked knowledge hiding to lower creativity and weaker innovation-related outcomes (Anand et al., 2022; Černe et al., 2014; Serenko and Bontis, 2016). Therefore, it is important to examine whether and how HPWS shape knowledge hiding in order to better understand how employees’ IWB can be enhanced.

To explain how HPWS are associated with knowledge hiding and IWB, this study integrates the AMO model (Appelbaum et al., 2000; Jiang et al., 2012), social exchange theory (Blau, 1964; Cropanzano and Mitchell, 2005), and goal orientation theory (Button et al., 1996; Dweck and Leggett, 1988). These theories explain different but connected stages of the process through which HPWS are associated with employee knowledge behavior and innovation. The AMO model specifies the resources and signals embedded in HPWS. Social exchange theory and goal orientation theory explain two interconnected interpretations of these organizational conditions: whether constructive knowledge behavior is relationally appropriate and whether it is instrumentally useful for achieving competence-demonstration goals. These interpretations are manifested behaviorally in employees’ decisions to hide or provide requested knowledge, and their behaviors subsequently affect the informational and relational resources needed for IWB.

Based on this integrated framework, the present study proposes that employee-perceived HPWS are positively associated with IWB and negatively associated with knowledge hiding, whereas knowledge hiding is negatively associated with IWB. Knowledge hiding represents the tested behavioral mechanism connecting HPWS with IWB. Performance goal orientation (PGO) functions as a boundary condition because it influences employees’ sensitivity to the achievement-related meaning of the HR environment. We therefore expect the negative HPWS–knowledge-hiding relationship, and consequently the positive indirect association between HPWS and IWB through reduced knowledge hiding to be stronger among employees high in PGO.

This study makes three contributions. First, it complements the conventional resource-enabling explanation of HPWS with a barrier-reduction explanation. HPWS may support innovation not only by adding capabilities and opportunities but also by reducing deliberate knowledge obstruction. Second, it integrates the AMO model, social exchange theory, and goal orientation theory into a coherent account of organizational provisions, relational and achievement-related interpretations, knowledge behavior, and innovation outcomes. Finally, it develops a context-sensitive explanation of PGO by proposing that performance-oriented employees’ knowledge behavior depends on whether the HR environment makes knowledge protection or constructive contribution a more viable route to competence demonstration.

## Theory and hypotheses

As mentioned previously, this study integrates the AMO model, social exchange theory, and goal orientation theory to explain how employees translate their perceptions of HPWS into knowledge-related behavior and IWB. Each framework addresses a distinct question within a connected theoretical process. The AMO framework addresses the question of what organizational conditions HPWS provide. Social exchange theory addresses why employees may respond to those conditions through constructive or defensive behavior. Goal orientation theory addresses why employees differ in their sensitivity to the achievement-related implications of the same conditions and in their strategy to demonstrate competence.

At the organizational-input stage, the AMO framework specifies the content of HPWS. Ability-enhancing practices, such as selective staffing, training, and developmental opportunities, strengthen employees’ competence and confidence in their capacity to acquire and apply knowledge. Motivation-enhancing practices, such as appraisal, rewards, recognition, and career opportunities, communicate the forms of effort and contribution valued by the organization. Opportunity-enhancing practices, such as participation, involvement, and open communication, provide channels through which employees can apply their capabilities, interact with coworkers, and make their contributions visible (Appelbaum et al., 2000; Jiang et al., 2012).

However, these practices do not determine employee behavior automatically. Employees interpret the meaning and purpose of HR practices before deciding how to respond (Nishii et al., 2008). Social exchange theory provides the principal relational explanation for this interpretation. When employees perceive that the organization invests in their development, evaluates them fairly, and provides meaningful opportunities to participate, they are more likely to interpret the employment relationship as supportive and experience an obligation to reciprocate through constructive behavior (Blau, 1964; Cropanzano and Mitchell, 2005). The target of reciprocal behavior need not be the specific organizational actor who originally provided a benefit. Because organizations function through interdependent relationships, employees may reciprocate organizational investment by assisting coworkers, contributing to shared objectives, and refraining from behavior that obstructs collective functioning. Responding constructively to a coworker’s request for knowledge can therefore constitute generalized organizational reciprocity. Knowledge hiding, by contrast, may violate the cooperative expectations implied by a supportive employment relationship.

Goal orientation theory complements this relational explanation by specifying how achievement goals influence employees’ interpretation of the same environment. Goal orientations are self-regulatory frameworks that influence how individuals define competence, attend to achievement-related information, and select strategies in evaluative situations (Dweck, 1986; Dweck and Leggett, 1988; DeShon and Gillespie, 2005). Employees do not merely decide whether to reciprocate organizational investment but also assess whether a particular behavior will help them maintain or demonstrate competence. The present study focuses on PGO as conceptualized by Button et al. (1996). This broad orientation captures employees’ concern with demonstrating competence and obtaining favorable evaluations. Employees high in PGO are especially attentive to cues concerning the visibility and evaluation of their competence. Knowledge-related behavior can become strategically relevant to such employees. Exclusive control over valuable knowledge may preserve an image of indispensability or distinctiveness. Alternatively, applying knowledge to solve problems and assist others may visibly demonstrate expertise. Goal orientation theory therefore does not predict that high-PGO employees will invariably hide knowledge. It predicts that they will be especially attentive to which behavioral strategy appears more effective for achieving favorable competence evaluations, a context-sensitive view consistent with recent evidence showing that the relationship between the narrower construct of performance-prove goal orientation and knowledge sharing varies across social and team contexts, including coworker popularity and team innovation climate (Chae and Shin, 2024; Lei, 2024).

HPWS can influence this strategy evaluation. Training and development reduce employees’ dependence on protecting their current stock of knowledge by signaling that competence can be renewed and expanded. Participation creates opportunities to demonstrate expertise through visible contribution. Fair appraisal and organizational support indicate that sustained contribution may be recognized by the organization (Appelbaum et al., 2000; Jiang et al., 2012). Repeated interaction also increases the reputational risks associated with intentional withholding. Strong HPWS can therefore make contribution a more viable competence-demonstration strategy and knowledge hiding a less attractive one.

In summary, the central psychological process underlying the model concerns employees’ evaluation of the relational appropriateness and achievement-related instrumentality of knowledge hiding. From a social exchange perspective, employees consider whether withholding knowledge is compatible with the reciprocity expected in the employment relationship. From a goal orientation perspective, they consider whether withholding or contributing provides a more useful strategy for demonstrating competence. Knowledge hiding and IWB are the behavioral outcomes of these interconnected evaluations.

### High-performance work systems and innovative work behavior

HPWS are widely regarded as an important organizational antecedent of IWB because they shape the conditions under which employees develop capabilities, direct effort, and act on new ideas. As a coherent bundle of mutually reinforcing HR practices, HPWS enhance employees’ ability, motivation, and opportunity to contribute beyond prescribed role requirements, thereby creating a work context that is more conducive to innovation (Appelbaum et al., 2000; Jiang et al., 2012). This is particularly important because IWB, as conceptualized by Janssen (2000), is not confined to the generation of novel ideas; rather, it encompasses a broader process of idea generation, idea promotion, and idea realization.

HPWS strengthen employees’ knowledge base, confidence, and work-related capabilities through training, developmental feedback, and continuous skill investment, thereby increasing their capacity to identify opportunities, integrate information, and generate useful solutions to work-related problems (de Jong and den Hartog, 2010; Jiang et al., 2012; Scott and Bruce, 1994). At the same time, performance-based rewards, recognition, and internal development opportunities channel employee effort toward behaviors valued by the organization, increasing employees’ willingness to invest effort in innovation-related activities rather than restricting themselves to routine task completion. In addition, HPWS create participative and resource-rich conditions that support innovation-related behavior. Practices such as employee involvement, decentralized decision-making, and information sharing broaden employees’ access to relevant knowledge and create opportunities to express suggestions, promote ideas, and contribute to work improvement (Miao et al., 2020; Tang et al., 2017). Because IWB includes idea promotion and realization as well as idea generation, such supportive conditions are particularly important.

From a social exchange perspective, HPWS also signal organizational support and investment. When employees perceive that the organization provides developmental opportunities, fair rewards, and participation channels, they are more likely to reciprocate through constructive and discretionary behaviors. IWB represents one such form of reciprocity because it requires employees to go beyond routine task performance by generating, promoting, and implementing new ideas. Taken together, HPWS enhance employees’ capacity to generate novel ideas, strengthen their motivation to pursue them, and provide the organizational space needed to promote and realize them. Accordingly, employees working in high-HPWS environments are more likely to engage in IWB.

*H1*: Employees’ perceptions of HPWS are positively related to IWB.

### The mediating role of knowledge hiding

Although HPWS can directly promote IWB, the behavioral mechanism underlying this relationship also deserves attention. Drawing on the AMO model and social exchange theory, we propose that one important mechanism is knowledge hiding. Prior research identifies fear of losing competitive advantage and distrust toward knowledge seekers as important motivations for knowledge hiding (Connelly et al., 2012). We argue that HPWS may weaken these motivations through ability-, motivation-, and opportunity-enhancing practices.

From an ability perspective, HPWS promote continuous skill development through training and learning opportunities (Appelbaum et al., 2000; Jiang et al., 2012). These practices enhance employees’ knowledge, skills, and abilities. Building on this premise, we argue that employees who perceive continued opportunities to develop their competence may be less likely to regard their existing knowledge as a scarce personal asset that must be protected to preserve individual indispensability. In this sense, ability-enhancing practices may reduce the self-protective reasoning underlying knowledge hiding (Connelly et al., 2019).

From a motivation perspective, HPWS align employee interests with collective goals through performance-based rewards, developmental appraisal, and internal career opportunities. Such practices signal that the organization values sustained contribution and collaboration rather than short-term individual advantage (Jiang et al., 2012). Under these conditions, the perceived benefits of withholding knowledge for personal gain become less attractive. More importantly, when HPWS emphasize cooperation and collective contribution, employees are encouraged to view helping coworkers and responding to knowledge requests as compatible with their own advancement. Research on knowledge hiding likewise suggests that cooperative and achievement-oriented climates reduce the tendency to conceal knowledge because employees are encouraged to regard contribution to collective success as consistent with personal success (Černe et al., 2014; El-Kassar et al., 2022). Importantly, this argument concerns HPWS as a coherent and mutually reinforcing system rather than isolated performance-contingent rewards. Although individual reward practices may sometimes intensify competition, a broader HPWS bundle that combines developmental support, participative opportunities, fair appraisal, and cooperation-supporting practices is more likely to signal that employee advancement is tied to sustained contribution and collaborative effectiveness rather than to zero-sum knowledge protection.

From an opportunity perspective, HPWS create structural conditions that facilitate interaction, participation, and information exchange (Appelbaum et al., 2000; Jiang et al., 2012). Through participative decision-making and open communication channels, employees are embedded in a relational environment characterized by trust, visibility, and reciprocity. Repeated participation and open communication may reduce uncertainty about coworkers’ intentions, making knowledge seekers appear less as opportunistic competitors and more as legitimate exchange partners. In such contexts, the social and relational costs of knowledge hiding become more salient (Connelly and Zweig, 2015; Cropanzano and Mitchell, 2005). Because intentionally withholding requested knowledge is more likely to be noticed and interpreted as a violation of cooperative norms, knowledge hiding becomes less socially acceptable and less strategically viable.

This logic is consistent with social exchange theory. When organizations invest in employees through developmental HR practices and create a cooperative work environment, they are more likely to reciprocate not only by sharing knowledge, but also by refraining from deliberately hiding requested knowledge from coworkers. Recent research also shows that knowledge hiding is shaped by organizational conditions, including HR practices, and carries important performance-related consequences (Anand et al., 2022; Shen et al., 2025). Therefore, we propose the following:

*H2*: Employees’ perceptions of HPWS are negatively related to knowledge hiding.

According to social exchange theory, workplace relationships are embedded in ongoing exchange processes through which individuals evaluate the benefits and costs of interacting with others. Over time, repeated exchanges cultivate trust, mutual obligation, and expectations of reciprocity among organizational members (Blau, 1964; Emerson, 1976; Homans, 1958). In this sense, reciprocity serves as a central normative basis for sustaining cooperative relationships in organizations.

In organizational contexts, knowledge sharing constitutes a particularly important form of social exchange because it depends heavily on trust and reciprocal willingness. When employees provide information, advice, or expertise to colleagues, they typically do so with at least an implicit expectation that such support will be returned when needed (Gouldner, 1960). Knowledge hiding disrupts this exchange logic. When an employee intentionally withholds requested knowledge, coworkers are likely to interpret this behavior as a violation of reciprocal expectations, thereby eroding trust and weakening the quality of exchange relationships (Connelly et al., 2012; Connelly and Zweig, 2015).

Such relational deterioration is likely to undermine IWB. IWB rarely emerges in isolation; rather, it depends on employees’ ability not only to generate novel ideas, but also to refine, promote, and implement those ideas in interaction with others (Janssen, 2000). In this regard, knowledge hiding constrains more than the creative stage of idea generation. More critically, it undermines the trust and cooperation needed to secure feedback, obtain support, mobilize resources, and coordinate implementation. When reciprocal exchange relationships are damaged by knowledge hiding, employees are less likely to access diverse perspectives, timely input, and the relational resources required to translate ideas into concrete outcomes (Černe et al., 2017; Connelly and Zweig, 2015).

Prior research has increasingly shown that knowledge hiding undermines IWB. Černe et al. (2017) found that knowledge hiding constrains employees’ IWB. Similarly, Mubarak et al. (2022) showed that leaders’ knowledge hiding is negatively associated with employees’ IWB. Taken together, knowledge hiding weakens reciprocal exchange relationships, restricts access to valuable knowledge resources, and undermines the trust and cooperation required to move ideas from generation to implementation. As a result, employees who engage in knowledge hiding are less likely to obtain the informational and relational resources needed to carry out IWB. Therefore, we propose:

*H3*: Knowledge hiding is negatively related to IWB.

The preceding arguments identify two complementary pathways connecting HPWS with IWB. The first is a resource-enabling pathway. HPWS directly enhance the abilities, motivation, and opportunities needed to generate, promote, and implement ideas. The second is a barrier-reduction pathway. HPWS reduce intentional knowledge hiding, thereby preserving the information access, reciprocity, and collaborative support required for innovation. The barrier-reduction pathway integrates the organizational, relational, and self-regulatory components of the theoretical framework. HPWS provide developmental resources, motivational signals, and participation opportunities. Employees interpret these provisions in terms of their exchange relationship with the organization and the strategies available for demonstrating competence. These interpretations influence whether employees respond constructively to knowledge requests or retain control over knowledge through concealment. Employees who refrain from hiding knowledge are better positioned to obtain information and feedback, gain colleagues’ endorsement of their ideas, and receive assistance during implementation. Because these resources and forms of social support are essential for engaging in IWB, HPWS should have a positive indirect association with IWB through reduced knowledge hiding.

*H4*: Knowledge hiding mediates the relationship between employees’ perceptions of HPWS and IWB.

### The moderating role of performance goal orientation

Employees may not respond uniformly to the organizational provisions embedded in HPWS. Goal orientation theory suggests that employees differ in how they define competence, interpret achievement situations, and select strategies for obtaining favorable evaluations (Dweck, 1986; Dweck and Leggett, 1988). PGO reflects a concern with demonstrating competence and receiving favorable evaluations. Employees high in PGO are especially attentive to information indicating whether their capabilities are visible, whether their performance compares favorably with relevant standards, and whether a particular behavior will protect or enhance their competence image (Button et al., 1996; DeShon and Gillespie, 2005).

Knowledge behavior becomes relevant to these concerns because knowledge can be used in different ways to demonstrate or protect competence. One possible strategy is knowledge protection. Employees may retain exclusive control over valuable knowledge because possessing expertise that others lack can preserve distinctiveness and perceived indispensability. A second strategy is constructive contribution. Employees may demonstrate competence by applying knowledge to problems, responding effectively to requests, and making visible contributions to collective outcomes. Goal orientation theory suggests that high-PGO employees will be especially attentive to which strategy appears likely to produce favorable evaluation in a particular environment.

When HPWS are weak, employees receive fewer consistent cues that continued development, participation, and constructive contribution will be supported. They may also have fewer opportunities to demonstrate expertise through visible involvement. Under these circumstances, exclusive control over knowledge may appear to be a comparatively useful way to protect a competence image. Employees high in PGO should be especially sensitive to this uncertainty and may therefore be more inclined to engage in knowledge hiding (Han et al., 2021; Sung et al., 2024). This reasoning is consistent with Zhu et al. (2019), who found that the relationship between performance-prove goal orientation and knowledge hiding depended on whether workplace conditions framed coworkers more as rivals or allies.

Strong HPWS alter the achievement context in several ways. Training and development communicate that employees can maintain their value by developing new competence rather than relying exclusively on existing expertise. Participation creates opportunities to make competence visible through problem solving and contribution. Fair appraisal and organizational support can indicate that constructive participation is a legitimate basis for recognition (Appelbaum et al., 2000; Jiang et al., 2012). Under strong HPWS, knowledge hiding may therefore become a less attractive and useful strategy for demonstrating competence. High-PGO employees should be particularly responsive to this change because they are more attentive to the achievement-related signals communicated by the organizational context (Button et al., 1996; DeShon and Gillespie, 2005). Strong HPWS do not remove their concern with favorable evaluation; instead, they can alter how that concern is behaviorally pursued. Employees low in PGO should be less preoccupied with demonstrating competence and obtaining favorable evaluations. HPWS may still reduce their knowledge hiding through capability development and exchange obligations, but the additional effect of changing the perceived strategy for competence demonstration should be less pronounced. Consequently, the difference in knowledge hiding between weak and strong HPWS conditions should be greater among employees high in PGO. This expectation is also consistent with broader evidence suggesting that knowledge hiding is shaped jointly by contextual and dispositional factors (Anand et al., 2022; Shen et al., 2025).

*H5*: PGO moderates the negative relationship between HPWS and knowledge hiding, such that this negative relationship is stronger when PGO is high.

Building on the preceding arguments, HPWS are expected to promote IWB indirectly by reducing knowledge hiding. HPWS are expected to reduce knowledge hiding by changing the capability, relational, and achievement-related conditions surrounding employees’ responses to knowledge requests. Reduced knowledge hiding, in turn, preserves the information access, feedback, and cooperative support required for IWB. Because employees high in PGO are particularly attentive to the achievement-related implications of organizational conditions, their knowledge-hiding behavior should respond more strongly to HPWS. When HPWS are weak, knowledge protection may appear to offer a useful means of protecting a favorable competence image. When HPWS are strong, competence development and constructive contribution become more viable routes to favorable evaluation, producing a more pronounced reduction in knowledge hiding among high-PGO employees. A greater reduction in knowledge hiding should preserve more of the reciprocal information exchange and interpersonal support needed for innovation. Consequently, the positive indirect association between HPWS and IWB through reduced knowledge hiding should become stronger as PGO increases. Therefore, we propose:

*H6*: PGO moderates the indirect effect of HPWS on IWB via knowledge hiding, such that the indirect positive effect is stronger when PGO is high.

## Materials and methods

### Participants and procedure

To test the proposed research model, data were collected from full-time employees working in China. A convenience sampling approach was employed, and an online questionnaire was distributed to employees from multiple organizations over a one-month period. Participation in the study was voluntary, and respondents were assured that their answers would remain anonymous and confidential. They were also informed that the data would be used exclusively for academic research purposes.

A total of 528 questionnaires were distributed. After excluding 72 questionnaires due to failed screening criteria, missing key variables, or inconsistent response patterns, 456 valid responses were retained for the final analysis, yielding a usable response rate of 86.36%. Participants were instructed to complete the survey based on their current work experience.

The final sample consisted of 240 men (52.63%) and 216 women (47.37%). In terms of age, 42.98% of respondents were between 18 and 30 years old, 31.58% were between 31 and 40, 17.11% were between 41 and 50, 7.02% were between 51 and 60, and 1.32% were over 60. Regarding educational attainment, 28.73% held a junior-college degree, 45.18% held a bachelor’s degree, 10.31% had completed high school, 13.38% held a master’s degree, and 2.41% held a doctoral degree.

Respondents came from diverse industry backgrounds, including information technology (18.4%), sales (16.7%), service-related sectors (15.8%), production/manufacturing (12.7%), finance (9.4%), education (9.4%), trade (7.7%), construction (7.2%), and legal services (2.6%). With respect to organizational position, 54.39% were general staff members, 25.88% were professional or technical employees, 14.04% held managerial positions, and 5.70% reported other positions. Organizational tenure was also diverse, with 15.79% reporting less than 1 year, 23.90% reporting 1–3 years, and 20.61% reporting 3–5 years, while the remainder reported longer tenure.

To provide additional geographic context, we summarized respondents’ IP-based survey locations. Respondents were distributed across 31 provincial-level regions in mainland China, including Anhui, Beijing, Chongqing, Fujian, Gansu, Guangdong, Guangxi, Guizhou, Hainan, Hebei, Heilongjiang, Henan, Hubei, Hunan, Inner Mongolia, Jiangsu, Jiangxi, Jilin, Liaoning, Ningxia, Qinghai, Shaanxi, Shandong, Shanghai, Shanxi, Sichuan, Tianjin, Tibet, Xinjiang, Yunnan, and Zhejiang. Because IP-based geographic information reflects respondents’ survey locations rather than independently verified firm locations, it was used only to describe respondents’ geographic distribution.

### Measures

All focal constructs were assessed using established scales from prior research. Prior to data collection, we reviewed Chinese-language and Chinese-context studies to identify existing translations, adaptations, or applications of the focal measures. This review indicated that the correspondence between existing Chinese versions and the original instruments varied across constructs. For IWB and knowledge hiding, prior Chinese employee studies have used measures closely aligned with Janssen’s (2000) nine-item IWB scale and Connelly et al.’s (2012) 12-item knowledge hiding scale. These studies were therefore used as important references for item wording and construct interpretation. For PGO and HPWS, the available Chinese measures were less directly aligned with the original instruments used in the present study. In our review, we did not identify Chinese employee-context applications that fully matched the original item composition, construct coverage, and validation context of the focal instruments. Accordingly, to preserve consistency with the theoretical definitions and item structures of the original scales while ensuring contextual appropriateness for employees working in China, the original instruments were retained as the primary basis, and existing Chinese versions were used as supplementary translation references.

To ensure linguistic and conceptual equivalence, we followed a forward–back translation procedure consistent with Brislin’s (1970) recommendations. The original English items were first translated into Chinese by bilingual researchers. An independent bilingual researcher then back-translated the Chinese items into English. The original and back-translated English versions were compared, and discrepancies were resolved through iterative discussion. During this process, existing Chinese item formulations from prior studies were consulted, particularly for the IWB and knowledge hiding measures. To further enhance contextual appropriateness, the translated questionnaire was reviewed by bilingual researchers and respondents familiar with Chinese workplace settings. Minor wording refinements were made to improve clarity and workplace relevance without altering the substantive meaning of the items.

All items were rated on a five-point Likert scale ranging from 1 = strongly disagree to 5 = strongly agree. Composite scores were calculated by averaging the relevant item responses. To provide evidence of measurement reliability and validity in the present sample, we assessed internal consistency reliability and conducted confirmatory factor analysis.

#### Performance goal orientation (PGO)

PGO was measured using a 10-item scale adapted from Button et al. (1996), which assesses individuals’ tendency to demonstrate competence and seek favorable evaluations in achievement-related settings. A representative item is “I prefer to do things that I can do well rather than things that I do poorly.” The scale showed high internal consistency reliability in the present study (Cronbach’s α = 0.889).

#### Learning goal orientation (LGO)

LGO was included as a scale-based control variable and was measured using a 10-item scale adapted from Button et al. (1996). The scale captures individuals’ tendency to develop competence through learning, effort, and challenging tasks. Representative items include “I prefer to work on tasks that force me to learn new things” and “The opportunity to do challenging work is important to me.” The scale showed high internal consistency reliability in the present study (Cronbach’s α = 0.885).

#### High-performance work systems (HPWS)

HPWS was measured using a 13-item scale adapted from Kroon et al. (2013), reflecting employees’ perceptions of HR practices associated with employee selection, training, performance appraisal, compensation, participation, job security, and organizational support. A representative item is “Our company provides employees with extensive training programs.” The scale demonstrated high internal consistency reliability in the present study (Cronbach’s α = 0.899).

#### Innovative work behavior (IWB)

IWB was measured using the 9-item scale developed by Janssen (2000), which captures employees’ engagement in idea generation, idea promotion, and idea realization. A representative item is “I generate new ideas for difficult issues.” The reliability of this scale was satisfactory in the present study (Cronbach’s α = 0.889).

#### Knowledge hiding

Knowledge hiding was measured using the 12-item scale developed by Connelly et al. (2012), which captures three forms of knowledge hiding: evasive hiding, playing dumb, and rationalized hiding. A representative item is “I pretended that I did not know the information requested by my colleague.” The scale showed high internal consistency reliability in the present study (Cronbach’s α = 0.888).

### Control variables

This study controlled for gender, age, education level, organizational tenure, and learning goal orientation (LGO). Gender, age, education level, and organizational tenure were included because these demographic and human capital characteristics may influence employees’ knowledge-related behaviors and IWB by reflecting differences in work experience, cognitive resources, organizational socialization, and workplace relationships. LGO was included to account for mastery-oriented motivational tendencies that are conceptually distinct from PGO. Controlling for LGO therefore allowed for a clearer estimation of the unique role of PGO as the focal moderator in the proposed model.

Other variables related to knowledge exchange and innovation, such as organizational trust and psychological safety, were not included as control variables in the main analyses. These factors are conceptually closer to potential mechanisms through which HPWS influence employee behavior than to exogenous background characteristics. Because HPWS are theorized to shape employees’ relational and psychological work conditions, controlling for organizational trust or psychological safety could remove part of the theoretically meaningful effect of HPWS and introduce overcontrol bias.

### Analytical strategy

Statistical analyses were conducted in several stages. First, descriptive statistics, reliability estimates, and zero-order correlations were computed using SPSS 26.0. Second, confirmatory factor analysis (CFA) was performed using AMOS 24.0 to test the validity of measurements. Third, hierarchical regression analyses were conducted to test the research hypotheses. Prior to creating the interaction term, HPWS and PGO were mean-centered. Finally, mediation and moderated mediation effects were examined using Hayes’ PROCESS macro (Model 7; Hayes, 2022) with 5,000 bootstrap samples to generate 95% bootstrap confidence intervals.

## Results

### Measurement validity and common method bias

Because all focal variables were collected from the same respondents using self-report measures, we assessed the potential influence of common method bias through both procedural and statistical remedies. As described in the Participants and Procedure section, participation was voluntary and the respondents were assured of anonymity and confidentiality and were informed that the data would be used solely for academic research purposes. These procedural safeguards were intended to reduce evaluation apprehension and encourage candid responses. As an initial statistical assessment, Harman’s single-factor test was conducted. The first unrotated factor accounted for 23.57% of the total variance, which is below the commonly used 50% heuristic threshold. This result suggests that no single factor accounted for the majority of the covariance among the measures. However, because Harman’s single-factor test alone is insufficient to rule out common method bias, additional statistical analyses were performed. Specifically, a common latent factor approach was used by adding an unmeasured latent factor loading on all observed variables and comparing the standardized factor loadings with and without the latent factor (Podsakoff et al., 2003). The differences in the factor loadings were all less than 0.20, indicating that common method bias was unlikely to be a serious concern.

Although hierarchical regression analyses were used to test the research hypotheses, confirmatory factor analysis (CFA) was conducted to assess the validity of the measurement model. Convergent validity was evaluated using standardized factor loadings, average variance extracted (AVE), and composite reliability (CR), whereas discriminant validity was examined by comparing AVE values with the squared inter-construct correlations (Fornell and Larcker, 1981).

Because the primary purpose of the measurement analysis was to establish construct validity and examine relationships among constructs rather than to evaluate item-level psychometric properties or develop a new measurement instrument, item parceling was considered appropriate (Little et al., 2002; Matsunaga, 2008). Since PGO was conceptualized as a unidimensional construct and previously validated as such, content-based parceling was not applicable. Therefore, random parceling was employed because previous simulation studies have shown that when items measure a single underlying factor, different parceling strategies, including random assignment, generally produce comparable structural parameter estimates while yielding a more parsimonious measurement model (Bandalos, 2002; Sass and Smith, 2006). In contrast, HPWS, knowledge hiding, and IWB were conceptualized as multidimensional constructs. Accordingly, content-based parceling was adopted so that each parcel represented a theoretically meaningful subdimension, thereby preserving the conceptual structure of the multidimensional constructs (Little et al., 2002).

The results of the analysis indicated a good model fit (χ^2^/df = 1.159, CFI = 0.997, TLI = 0.996, RMR = 0.018, RMSEA = 0.019). As shown in Table 1, all standardized factor loadings exceeded 0.60, CR values were greater than the recommended threshold of 0.70, and the AVE for each construct exceeded the conventional cutoff of 0.50, demonstrating adequate convergent validity. Furthermore, the AVE for each construct exceeded the squared correlations among the constructs, providing evidence of discriminant validity (Fornell and Larcker, 1981).

**Table 1:** Confirmatory factor analysis results.

Latent variable	Observed variable	Standardized factor loadings	AVE	CR
HPWS	Ability	0.835***	0.716	0.883
	Motivation	0.859***		
	Opportunity	0.845***		
IWB	Idea generation	0.761***	0.573	0.801
	Idea promotion	0.755***		
	Idea realization	0.754***		
PGO	PGO1	0.884***	0.724	0.887
	PGO2	0.835***		
	PGO3	0.833***		
KH	Evasive hiding	0.756***	0.578	0.804
	Playing dumb	0.778***		
	Rationalized hiding	0.746***		

***p < 0.001. HPWS, High-Performance Work Systems; IWB, Innovative Work Behavior; PGO, Performance Goal Orientation; KH, Knowledge Hiding.

### Descriptive statistics and correlation analysis

Means, standard deviations, and Pearson correlations among all variables are presented in Table 2. PGO was positively correlated with knowledge hiding (*r* = 0.364, *p* < 0.001) and negatively correlated with IWB (*r* = −0.350, *p* < 0.001). PGO was also positively associated with HPWS (*r* = 0.221, *p* < 0.001). HPWS was negatively correlated with KH (*r* = −0.363, *p* < 0.001) and positively correlated with IWB (*r* = 0.376, *p* < 0.001). In addition, KH was negatively correlated with IWB (*r* = −0.547, *p* < 0.001). Overall, these correlations were generally consistent with the proposed theoretical relationships and provided preliminary support for the hypotheses (Figure 1).

**Table 2:** Descriptive statistics and bivariate correlations.

Variable	M	SD	1	2	3	4	5	6	7	8	9
1. Gender	—	—	—								
2. Age	3.776	1.178	−0.066	—							
3. Education	2.689	0.914	0.045	−0.044	—						
4. Tenure	3.261	1.672	−0.011	0.582***	−0.083	—					
5. LGO	3.639	0.757	0.021	−0.024	0.112*	−0.087	—				
6. PGO	3.134	0.812	0.030	−0.044	0.009	0.016	−0.099*	—			
7. HPWS	3.492	0.750	0.096*	0.001	0.038	−0.039	0.325***	0.221***	—		
8. KH	2.385	0.737	−0.074	0.036	0.017	0.107*	−0.352***	0.364***	−0.363***	—	
9. IWB	3.480	0.818	0.096*	−0.035	0.000	−0.093*	0.420***	−0.350***	0.376***	−0.547***	—

*N* = 456. Gender (0 = male, 1 = female). *p < 0.05, ***p < 0.001. LGO, learning goal orientation; PGO, performance goal orientation; HPWS, high-performance work systems; KH, knowledge hiding; IWB, innovative work behavior.

**Figure 1:**
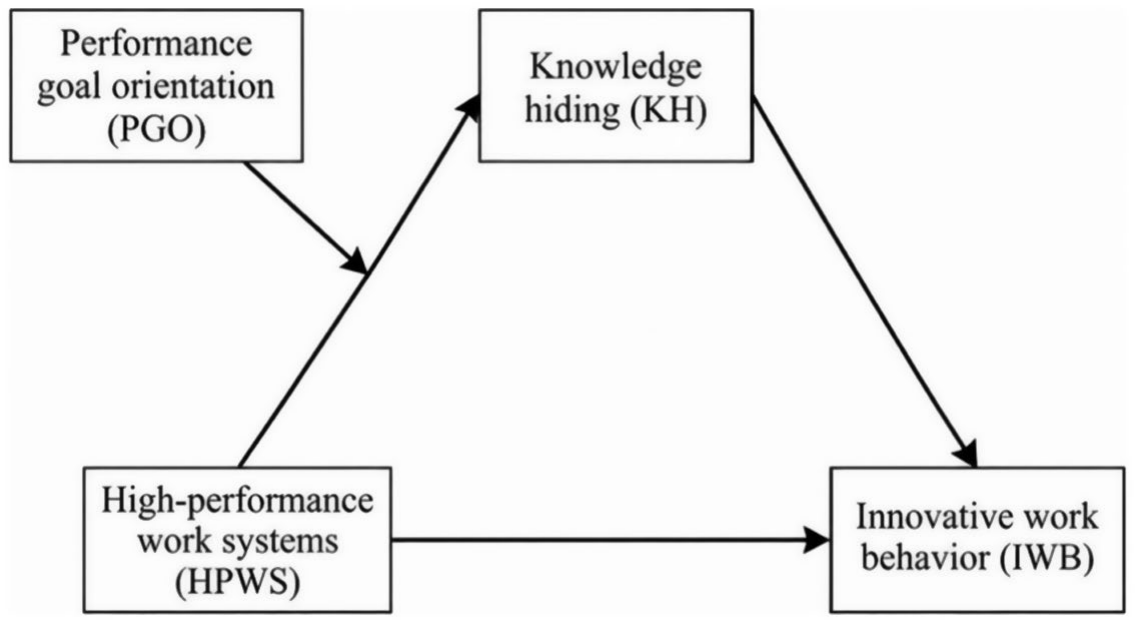
Conceptual model of the study.

### Hypothesis testing

Table 3 reports the hierarchical regression results for knowledge hiding. The VIF values ranged from 1.016 to 1.538, and all values were below the commonly recommended threshold of 5, indicating that multicollinearity was not a serious concern. In Model 1, only the control variables were entered. LGO was significantly and negatively associated with knowledge hiding (b = −0.340, *p* < 0.001), whereas gender, age, education, and organizational tenure were not significant predictors. The control variables explained 13.9% of the variance in knowledge hiding.

**Table 3:** Results of hierarchical regression analysis on knowledge hiding.

Knowledge hiding										
Variable	Model 1			Model 2			Model 3			VIF
	*B*	SE	*t*	*b*	SE	*t*	*b*	SE	*t*	
Gender	−0.104	0.065	−1.608	−0.068	0.055	−1.232	−0.065	0.055	−1.171	1.018
Age	−0.021	0.034	−0.618	0.007	0.029	0.250	0.004	0.029	0.132	1.534
Education	0.053	0.036	1.493	0.045	0.030	1.493	0.044	0.030	1.467	1.021
Tenure	0.045	0.024	1.867	0.029	0.020	1.437	0.030	0.020	1.475	1.538
LGO	−0.340	0.043	−7.911***	−0.175	0.039	−4.438***	−0.184	0.039	−4.684***	1.186
HPWS	—	—	—	−0.389	0.040	−9.606***	−0.385	0.040	−9.580***	1.219
PGO	—	—	—	0.394	0.035	11.120***	0.386	0.035	10.920***	1.103
HPWS × PGO	—	—	—	—	—	—	−0.115	0.043	−2.667**	1.016
R^2^	0.139***			0.376			0.386			
ΔR^2^	—			0.237***			0.010**			

*N* = 456. **p < 0.01, ***p < 0.001. LGO, learning goal orientation; HPWS, high-performance work systems; PGO, performance goal orientation. VIF values are from the final model.

In Model 2, HPWS and PGO were added to the regression model. HPWS was significantly and negatively related to knowledge hiding (b = −0.389, *p* < 0.001), thereby supporting H2. PGO was significantly and positively associated with knowledge hiding (b = 0.394, *p* < 0.001). The addition of HPWS and PGO substantially increased the explained variance from 13.9 to 37.6% (ΔR^2^ = 0.237, *p* < 0.001).

In Model 3, the interaction term between HPWS and PGO was entered. The interaction effect was significant (b = −0.115, *p* < 0.01), indicating that PGO moderated the relationship between HPWS and knowledge hiding. The inclusion of the interaction term explained additional variance in knowledge hiding (ΔR^2^ = 0.010, *p* < 0.01). As illustrated in Figure 2, the negative relationship between HPWS and knowledge hiding was stronger when PGO was high than when PGO was low. Thus, H5 was supported.

**Figure 2:**
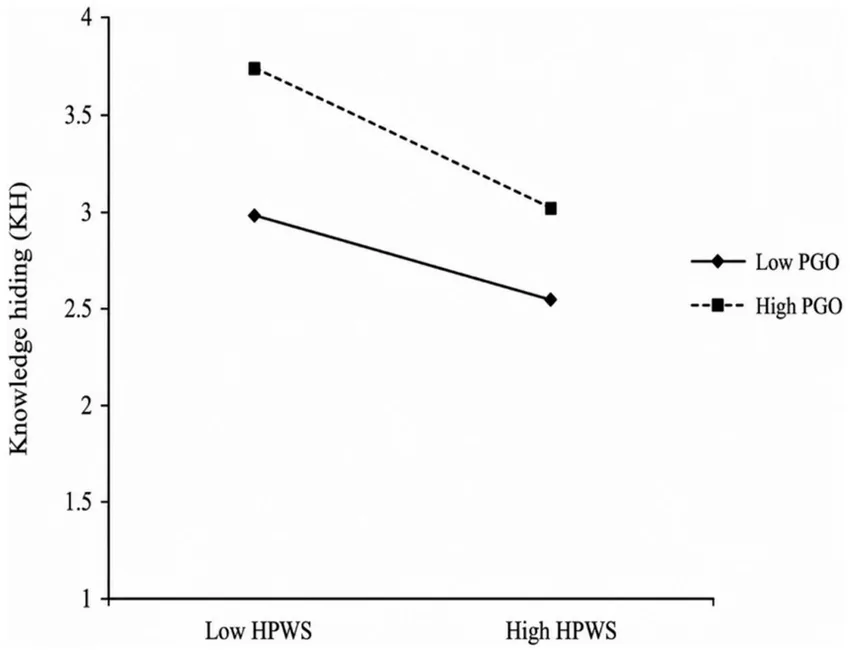
Moderating effect of performance goal orientation on the relationship between high-performance work systems and knowledge hiding.

Table 4 presents the hierarchical regression results for IWB. The VIF values ranged from 1.020 to 1.545, and all values were below the commonly recommended threshold of 5, indicating that multicollinearity was not a serious concern. In Model 1, gender was significantly and positively related to IWB (b = 0.148, *p* < 0.05). LGO was also significantly and positively associated with IWB (b = 0.453, *p* < 0.001). Age, education, and organizational tenure were not significant predictors. The control variables explained 19.1% of the variance in IWB.

**Table 4:** Results of hierarchical regression analysis on IWB.

Innovative work behavior							
Variable	Model 1			Model 2			VIF
	*B*	SE	*t*	*b*	SE	*t*	
Gender	0.148	0.070	2.123*	0.079	0.061	1.281	1.020
Age	0.015	0.036	0.404	0.002	0.032	0.063	1.524
Education	−0.050	0.038	−1.299	−0.025	0.034	−0.756	1.026
Tenure	−0.035	0.026	−1.379	−0.013	0.023	−0.586	1.545
LGO	0.453	0.046	9.777***	0.247	0.044	5.570***	1.223
HPWS	—	—	—	0.164	0.045	3.650***	1.222
KH	—	—	—	−0.450	0.046	−9.730***	1.257
R^2^	0.191***			0.381			
ΔR^2^	—			0.190***			

*N* = 456. *p < 0.05, ***p < 0.001. LGO, learning goal orientation; HPWS, high-performance work systems; KH, knowledge hiding. VIF values are from the final model.

In Model 2, HPWS and knowledge hiding were added to the regression model. The explanatory power of the model increased from 19.1 to 38.1% (ΔR^2^ = 0.190, *p* < 0.001). HPWS was significantly and positively related to IWB (b = 0.164, *p* < 0.001), thereby supporting H1. Knowledge hiding was significantly and negatively related to IWB (b = −0.450, *p* < 0.001), thereby supporting H3.

Finally, Table 5 presents the index of moderated mediation. The index was significant (index = 0.0516, Boot SE = 0.0200, 95% CI = [0.0121, 0.0919]), indicating that the indirect effect of HPWS on IWB through knowledge hiding varied significantly across levels of PGO. As shown in Table 6, the bootstrap 95% confidence intervals of the indirect effects of HPWS on IWB via knowledge hiding excluded zero regardless of values of PGO ([0.083, 0.184] at low PGO; [0.126, 0.223] at mean PGO, [0.155, 0.291] at high PGO), thereby supporting H4. The conditional indirect effects became stronger as PGO increased (0.130 at low PGO; 0.172 at mean PGO; 0.218 at high PGO), thereby supporting H6.

**Table 5:** Index of moderated mediation.

Moderator	Mediator	Estimate	Boot SE	Boot LLCI	Boot ULCI
PGO	Knowledge Hiding	0.052	0.020	0.012	0.092

5,000 bootstrap samples were extracted. PGO, Performance Goal Orientation; Boot SE, bootstrap standard error; LLCI, lower limit of 95% bootstrap confidence interval; ULCI, upper limit of 95% bootstrap confidence interval.

**Table 6:** Conditional indirect effects of HPWS on IWB via KH.

PGO	Effect	Boot SE	Boot LLCI	Boot ULCI
2.32	0.130	0.026	0.083	0.184
3.13	0.172	0.025	0.126	0.223
3.95	0.218	0.035	0.155	0.291

*N* = 456. Conditional indirect effects were estimated using Hayes’ PROCESS Model 7 with 5,000 bootstrap samples. Low, mean, and high levels of PGO correspond to one standard deviation below the mean, the mean, and one standard deviation above the mean, respectively. Boot SE, bootstrap standard error; Boot LLCI, lower limit of 95% bootstrap confidence interval; Boot ULCI, upper limit of 95% bootstrap confidence interval.

## Discussion

### Theoretical implications

This study offers several theoretical implications for research on strategic human resource management, knowledge hiding, goal orientation, and employee innovation. First, it contributes to the HPWS–innovation literature by complementing established resource-enhancing explanations with a barrier-reduction perspective. Prior research has linked HPWS to organizational and employee outcomes and has explained these relationships through employees’ abilities, motivation, participation opportunities, human capital, knowledge exchange, and discretionary contribution (Appelbaum et al., 2000; Collins and Smith, 2006; Huselid, 1995; Jiang et al., 2012; Kehoe and Wright, 2013). Employees’ perceptions of HPWS have been linked to IWB through exploratory learning, while organization-level HPWS have been linked to IWB through employee voice (Escribá-Carda et al., 2017; Miao et al., 2020).

Prior research has also connected HR practices with reduced knowledge hiding and innovation-related outcomes (El-Kassar et al., 2022). Recent evidence further indicates that perceived HPWS may foster knowledge sharing through perceived organizational support (Jeong et al., 2024) and may have positive and negative indirect associations with IWB through harmonious and obsessive passion, respectively (Farooqi et al., 2025). Extending this literature, the present study focuses on employee-perceived HPWS as a coherent system and identifies an indirect association with IWB via lower knowledge hiding. The findings are therefore consistent with a barrier-reduction pathway in which HPWS may support innovation not only by strengthening employee resources and contributions but also by weakening deliberate knowledge obstruction. This barrier-reduction pathway differs from commonly examined positive mediating mechanisms because knowledge hiding captures a deliberate behavioral obstruction of knowledge exchange rather than merely the absence of supportive attitudes or motivation.

Second, this study advances knowledge-hiding research by positioning knowledge hiding as a behavioral mechanism linking employee-perceived HR systems to innovation-related outcomes. Knowledge hiding differs from the mere absence of knowledge sharing because it involves an intentional attempt to withhold or conceal requested knowledge (Connelly et al., 2012; Connelly and Zweig, 2015). This distinction is relevant because knowledge hiding may constrain IWB depending on the surrounding motivational climate and job characteristics (Černe et al., 2017). The observed indirect association suggests that HPWS may support IWB partly by weakening a deliberate form of knowledge obstruction rather than only by encouraging knowledge sharing.

Third, this study refines the theoretical understanding of PGO by highlighting the context dependence of its behavioral implications. Button et al. (1996) conceptualized PGO in terms of demonstrating competence and obtaining favorable evaluations. Related research on the narrower construct of performance-prove goal orientation shows that its relationship with knowledge hiding depends on whether performance feedback emphasizes individual or group performance (Zhu et al., 2019). The present study extends, rather than directly replicates, this logic using the broader PGO construct. One possible interpretation of the observed interaction is that, when employees perceive strong developmental, participation, and information-exchange cues from HPWS, cooperative contribution may become a viable route through which high-PGO employees demonstrate competence. Thus, PGO should not necessarily be treated as uniformly detrimental; its behavioral expression may depend on whether contextual cues direct achievement concerns toward interpersonal rivalry or constructive contribution.

Finally, this study contributes to multi-theory integration by clarifying the complementary roles of social exchange theory, the AMO model, and goal orientation theory. Social exchange theory provides the overarching reciprocity logic linking perceived organizational investment with less defensive withholding and more constructive behavior (Blau, 1964; Cropanzano and Mitchell, 2005). The AMO framework specifies the HR-practice content through which HPWS enhance employees’ abilities, motivation, and opportunities to participate and exchange knowledge (Appelbaum et al., 2000; Jiang et al., 2012). Goal orientation research provides a rationale for why these HR cues may have different implications for employees with different levels of PGO (Button et al., 1996). By assigning these perspectives distinct but connected roles, the study offers a more coherent account of the association between HPWS, knowledge hiding, and IWB and of the conditions under which the indirect association becomes stronger.

### Practical implications

This study also offers several practical implications for organizations seeking to promote employee innovation. First, managers should recognize that HPWS may support innovation not only by enhancing employees’ skills and motivation, but also by reducing defensive knowledge behaviors. Therefore, HR practices should be designed to create a work environment in which knowledge contribution is safe, visible, and valued. For example, training and development programs can be used not only to improve employees’ task-related skills, but also to encourage collaborative problem solving and confidence in knowledge exchange. Similarly, participative decision-making, open communication channels, and developmental feedback may help employees view responding to knowledge requests as part of normal work contribution rather than as a threat to their personal advantage.

Second, organizations should pay close attention to the signals created by performance appraisal and reward systems. If evaluation systems emphasize only individual ranking, short-term achievement, or direct comparison among coworkers, employees may be more likely to regard knowledge as a private resource that protects their relative advantage. To reduce this risk, managers should consider balancing individual performance criteria with indicators that recognize cooperative contribution, knowledge responsiveness, team problem solving, mentoring, and support for others’ innovative efforts. Such practices may help employees pursue favorable evaluations through constructive contribution rather than defensive knowledge concealment.

Third, the findings suggest that employees with high PGO should not be treated simply as problematic or overly competitive. Because these employees are sensitive to competence demonstration and favorable evaluation, they may respond strongly to HR systems that define cooperation as a valued and visible form of performance. Managers can channel high-PGO employees’ achievement concerns by involving them in improvement projects, cross-functional problem solving, knowledge-sharing routines, or team-based innovation tasks where cooperative contribution can be recognized. In this way, performance-oriented motivation may be redirected away from interpersonal rivalry and knowledge protection toward constructive contribution.

Fourth, the findings suggest that managers should prioritize feasible practices that are theoretically consistent with reducing knowledge hiding and supporting innovation. These include transparent communication of performance expectations, fair and developmental feedback, opportunities for employee voice, recognition of knowledge contribution, and regular team-based problem-solving routines. Although the present study did not compare the relative effectiveness of specific HPWS practices, these practices are consistent with the broader logic that employees are less likely to hide knowledge when they perceive the work environment as developmental, participative, fair, and cooperation-oriented.

Overall, the practical implication of this study is that innovation management should not focus only on encouraging employees to generate new ideas. Organizations also need to reduce defensive behaviors that prevent knowledge from being exchanged, refined, and implemented. A coherent HR system that combines developmental support, fair evaluation, participation opportunities, and cooperation-oriented recognition may help create a workplace in which employees are less inclined to hide knowledge and more willing to contribute to IWB.

### Limitations and future research

This study has several limitations that should be acknowledged. First, the cross-sectional survey design limits strong causal inference. Although the hypothesized relationships were developed based on prior theory and empirical research, the temporal ordering among HPWS, knowledge hiding, PGO, and IWB cannot be firmly established. Reverse causality and alternative explanations therefore cannot be fully ruled out. Accordingly, the findings should be interpreted as associations that are consistent with the proposed theoretical model rather than as definitive evidence of causality. Future research should employ longitudinal, time-lagged, multi-wave, or experimental designs to examine causal ordering more rigorously and provide stronger evidence for the proposed moderated mediation process.

Second, the anonymous individual-level survey design and convenience sampling approach limit the representativeness of the sample and the generalizability of the findings. Respondents were recruited from multiple organizations and reported diverse industry backgrounds, while IP-based information indicated geographic dispersion across China. However, the study did not collect independently verifiable organizational identifiers or other firm-level characteristics, and respondents’ IP locations should not be interpreted as verified organizational locations. Accordingly, the findings should be interpreted as employee-level evidence from respondents working in China, and caution is warranted when generalizing them to specific organizational contexts. Future research should employ organization-based sampling and independently verified organizational data to examine whether the proposed relationships vary across industries, ownership forms, and regions.

Third, this study examined HPWS as an overall system based on employees’ perceptions rather than separating the effects of specific HR practices or HPWS configurations. This approach is consistent with the view of HPWS as a coherent bundle of mutually reinforcing practices, but it does not reveal whether particular practices are more strongly associated with reduced knowledge hiding and IWB. Future research could examine the relative and combined effects of specific HPWS practices to identify which elements are especially important for reducing defensive knowledge behaviors and supporting employee innovation.

Fourth, this study focused on PGO as the focal individual boundary condition. Although PGO is theoretically relevant because it captures employees’ sensitivity to evaluation, competence demonstration, and relative standing, other motivational orientations may also shape how employees interpret and respond to HPWS. Future research could examine learning goal orientation, performance-avoidance orientation, or other motivational dispositions to clarify whether different forms of achievement motivation lead to different knowledge-related and innovation-related responses.

Finally, this study relied on self-reported measures, particularly employees’ perceptions of HPWS and IWB. Although procedural remedies and statistical checks were used to reduce and assess common method bias, self-report data may still be affected by perceptual consistency, social desirability, or subjective interpretations of workplace practices. Future research could strengthen the robustness of the findings by incorporating multi-source and multi-level data, such as supervisor-rated IWB, coworker-rated knowledge hiding, or organization-level assessments of HPWS.

## Data Availability

The raw data supporting the conclusions of this article will be made available by the authors, without undue reservation.
